# Optimization of Mesoporous Carbon Adsorbents from Sucrose and Aerosil 380 Hydrophilic Silica by Hard Templating

**DOI:** 10.1021/acsomega.5c04653

**Published:** 2025-08-18

**Authors:** João Baptista, Ricardo Schneider, Kelen Menezes Flores Rossi de Aguiar, Aparecido Nivaldo Módenes, Fabiano Bisinella Scheufele

**Affiliations:** † Federal University of Technology—Paraná—UTFPR, Graduate Program in Chemical and Biotechnological Processes (PPGQB), Rua Cristo Rei, 19, Vila Becker, Toledo 85902-490, Paraná, Brazil; ‡ Group of Polymers and Nanostructures(GPAN), Federal University of Technology—Paraná—UTFPR, Rua Cristo Rei, 19, Vila Becker, Toledo 85902-490, Paraná, Brazil; § Chemical Engineering Department, Materials Laboratory, Western Paraná State University, Rua da Faculdade, 645, Jd. La Salle, Toledo 85903-000, Paraná, Brazil

## Abstract

Mesoporous carbon materials were synthesized by using sucrose as a carbon source and hydrophilic Aerosil 380 as a hard template. A two-stage optimization process based on the response surface methodology using a central composite design (RSM-CCD) was employed to enhance the adsorption performance of the material for the crystal violet (CV) dye. The first stage of optimization yielded a maximum adsorption capacity of 155.4 mg g^–1^ under carbonization conditions of 800 °C, 18.41 °C min^–1^, and 60 min. Further, optimization of sucrose (23% m/V) and silica template (17.07% m/V) concentrations led to a significantly higher capacity of 223.5 mg g^–1^. Characterization techniques confirmed the formation of amorphous graphitic structure (XRD), thermal decomposition of the organic phase near 350 °C (TGA-DTG), and effective Aerosil 380 removal confirmed by EDS and LIBS. Morphological and structural analyses using scanning electron microscopy (SEM) and high-resolution transmission electron microscopy (HRTEM) revealed a disordered mesoporous structure with turbostratic carbon layers. The optimized carbon exhibited a hierarchical mesoporous structure, with an *A*_total_ of 607.8 m^2^ g^–1^, *V*_total_ of 1.458 cm^3^ g^–1^, and an average *D*_p_ of 9.6 nm, demonstrating strong potential for micropollutant adsorption applications.

## Graphical abstract

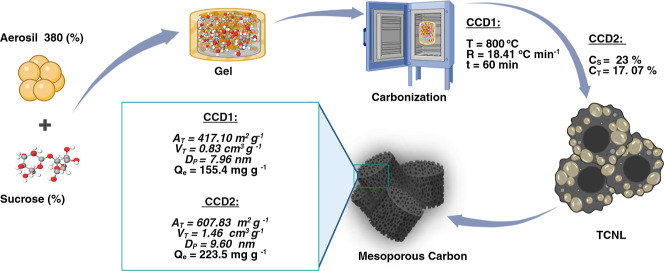

## Introduction

Mesoporous carbons are widely studied due to their various scientific applications, such as adsorbents, catalysts, gas storage, electrode materials, and drug delivery.^1–4^ According to the IUPAC classification, these materials have pores ranging from 2 to 50 nm^5^ being the pore network ordered^6,7^ or disordered,^8^ depending on the synthesis method and template used.

Mesoporous carbon materials can be synthesized by activation,^9^ catalytic,^10^ and templating methods. Unlike traditional activation methods, the template method can be preferable for the synthesis of mesoporous carbon since it allows better control of pore size, carbon morphology, and reproducible porous properties. This method can be divided into soft and hard template.^11^

The soft templating approach for mesoporous carbon synthesis employs organic surfactants or polymers as sacrificial templates. During carbonization, these compounds decompose, forming a well-defined mesoporous carbon framework.^12–15^ Cationic surfactants such as cetyltrimethylammonium bromide (CTAB) have been used to synthesize ordered mesoporous carbon.^16^ A nonionic copolymer surfactant, Pluronic F-127, has been employed to produce mesoporous carbon microspheres through hydrothermal carbonization.^17^ However, soft templating methods often suffer from limitations in the thermal stability, structural regularity, and reproducibility. In contrast, the hard templating method is widely regarded as more effective because it uses rigid solids such as silica,^7,18^ alumina,^19,20^ or zeolites^21–23^ to guide mesoporous carbon formation. These templates offer precisely tunable and highly defined pore networks and greater thermal stability compared to organic carbon precursors. Typically, the synthesis involves impregnating the template with a carbon source, followed by carbonization to create a rigid carbon matrix with an inverted pore structure. The template is then removed via chemical leaching, yielding the final mesoporous carbon.^24,25^ Due to its superior control over porosity, morphology, and structural robustness, hard templating remains the preferred method for applications requiring highly tailored mesoporous carbon materials.

Sucrose, a commonly used carbon precursor, is preferred for its wide availability and affordability.^26^ It allows for the formation of various morphologies and textural properties, depending on the template used. Some successful studies using sucrose with silica templates have reported high-surface areas.^18,27^ In addition, sucrose has been shown to generate mesoporous carbon with hierarchical architectures and adjustable pore size distributions when processed under appropriate conditions.^28^ These characteristics make sucrose a versatile carbon source for producing tailored mesoporous structures suitable for applications such as adsorption, catalysis, and energy storage.

However, many of these investigations applied fixed carbonization parameters without statistically correlating synthesis variables with the resulting structural and adsorptive properties. For instance, Schutjajew et al.^18^ varied sucrose/Aerosil 380 ratios (1:1, 1:2, 1:4), with 1:1 and 1:2 achieving higher surface areas (1230 and 987 m^2^ g^–1^) and pore volumes (0.17 and 0.22 cm^3^ g^–1^), compared to 1:4 (567 m^2^ g^–1^ and 0.15 cm^3^ g^–1^) under fixed carbonization (900 °C for 2 h). Besinela et al .^49^ observed higher-surface area and pore volume for 1.8:1 (462 m^2^ g^–1^ and 1.04 cm^3^ g^–1^) than 2.7:1 (244 m^2^ g^–1^ and 0.68 cm^3^ g^–1^) at constant conditions (700 °C for 1 h). Despite valuable findings, neither study included the statistical validation of textural trends. These examples highlight the need for a statistically guided and reproducible approach to correlate the synthesis parameters with material performance.

In this context, this study aims to synthesize mesoporous carbon using sucrose as a carbon source and hydrophilic fumed Aerosil 380 as a hard template. A statistically guided approach based on the response surface methodology (RSM) and central composite design (CCD) was employed to optimize the synthesis conditions and enhance the structural performance of the material. The resulting carbons were evaluated for their adsorption capacity using a crystal violet dye as a model contaminant. To comprehensively characterize the materials, a range of physical, chemical, and morphological analyses was conducted, providing insight into the relationship between synthesis parameters and adsorptive behavior.

## Results and Discussion

### Optimization of the Mesoporous Carbons Synthesis

Surface response methodology (RSM) with central composite design (CCD) was used in two successive steps to identify the most suitable conditions to produce mesoporous carbons (MC). The first step (CCD1) evaluated the impact of thermochemical parameters in the carbonization step (i.e., temperature (*T*), rate (*R*), and time (*t*)), while the second step (CCD2) examined the effects of concentrations of the template (*C*_T_ ) and carbon precursor (sucrose) (*C*_S_).

#### CCD1: Effect of the Thermal Parameters of Carbonization

The results of CCD1 for CV adsorption capacities as a function of carbonization variables (*T*, *R*, and *t*) are shown in Table 1. The results of individual experiments were obtained in duplicate (see Table S1 in the Supporting Information). Examining Table 1, it is evident that the interdependence of temperature, heating rate, and time during calcination plays a crucial role in determining the adsorption capacity (*Q*_e_) of MC. Optimal adsorption occurs within intermediate temperature ranges (600–800 °C), whereas higher temperatures (1000 °C) tend to reduce *Q*_e_, probably due to structural changes in the adsorbent. A higher heating rate, such as 15 °C min^–1^, generally improves *Q*_e_, but its effect is not uniform in all temperature ranges. Calcination time also significantly influences adsorption, although its impact is nonlinear; both shorter and longer durations can result in high adsorption, depending on the interdependence of other factors. Ultimately, optimal adsorption performance is achieved by carefully balancing these parameters, with the highest adsorption capacity observed in Run 15 (*T* = 800 °C, *R* = 18.41 °C min^–1^, and *t* = 60 min), as shown in Table 1.

**Table 1:** CCD1 Matrix: Effect of the Thermal Parameters of Carbonization onto CV Adsorption Capacity

run	*T* (°C)	*R* (°C min^–1^)	*t* (min)	*Q*_e_ (mg g^–1^)	s.d.^a^
1	600	5	30	89.704	5.563
2	1000	5	30	48.985	2.019
3	600	15	30	138.305	4.586
4	1000	15	30	59.957	0.839
5	600	5	90	129.234	1.970
6	1000	5	90	121.458	5.375
7	600	15	90	119.337	0.013
8	1000	15	90	50.530	3.313
9	800	10	60	67.337	5.284
10	800	10	60	89.781	5.524
11	800	10	60	61.878	3.932
12	463.64	10	60	109.764	0.355
13	1136.37	10	60	119.357	4.716
14	800	1.59	60	117.459	5.324
15	800	18.41	60	155.453	3.456
16	800	10	9.55	129.576	2.263
17	800	10	110.45	97.172	2.562

a s.d.: standard deviation.

An examination of MC crystal violet dye adsorption was performed in relation to carbonization thermal parameters, as demonstrated by the variance (ANOVA) results and the analysis of estimated effects (see Tables S2 and S3—Supporting Information), respectively. The significant effects highlighted in the variance analysis for CCD1, as shown in the Pareto chart (see Figure S1, Supporting Information), encompass the interaction between the heating rate and time (2L by 3L), the quadratic and linear terms of the heating rate (*Q*, *L*), and the temperature (*Q*) and the linear term of time (*L*); these results are confirmed with 95% confidence. In addition, these variables greatly influence the adsorption behavior of the crystal violet dye. The experiment yielded results that enabled the fitting of a quadratic model (eq 1), which was then used to determine the influence of the carbonization conditions (i.e., temperature (*T*), heating rate (*R*), and time (*t*)) on the CV adsorption capacity (*Q*_e_).

1 $$\begin{array}{l} Q_{e}\left({mg\;g}^{-1}\right)=179.596-0.364T-0.457R+1.682t-9.858\times 10^{-6}TR-0.00142Tt-0.209Rt\\ +0.000262T^{2}+0.7558R^{2}+0.0112t^{2} \end{array}$$

The quadratic model was validated by ANOVA and the *F*-test (see Table S2 in the Supporting Information), respectively. The experimental adsorption capacity of Run 15 was 155.453 mg g^–1^, while the value calculated by the quadratic model for the same conditions was 139.782 mg g^–1^, reaching a relative deviation of 10.08%. The response surfaces for the effect of the thermochemical carbonization parameters of the MC synthesis on the adsorption capacity of the CV dye are illustrated in Figure 1. The interaction between time and heating rate was significant and exhibited an antagonistic effect, while the interaction between time and temperature in Figure 1b also showed an antagonistic effect but was statistically insignificant. Nonetheless, the quadratic parameters are noteworthy and contribute to the 95% confidence level (see Figure S1 in the Supporting Information).

**Figure 1:**
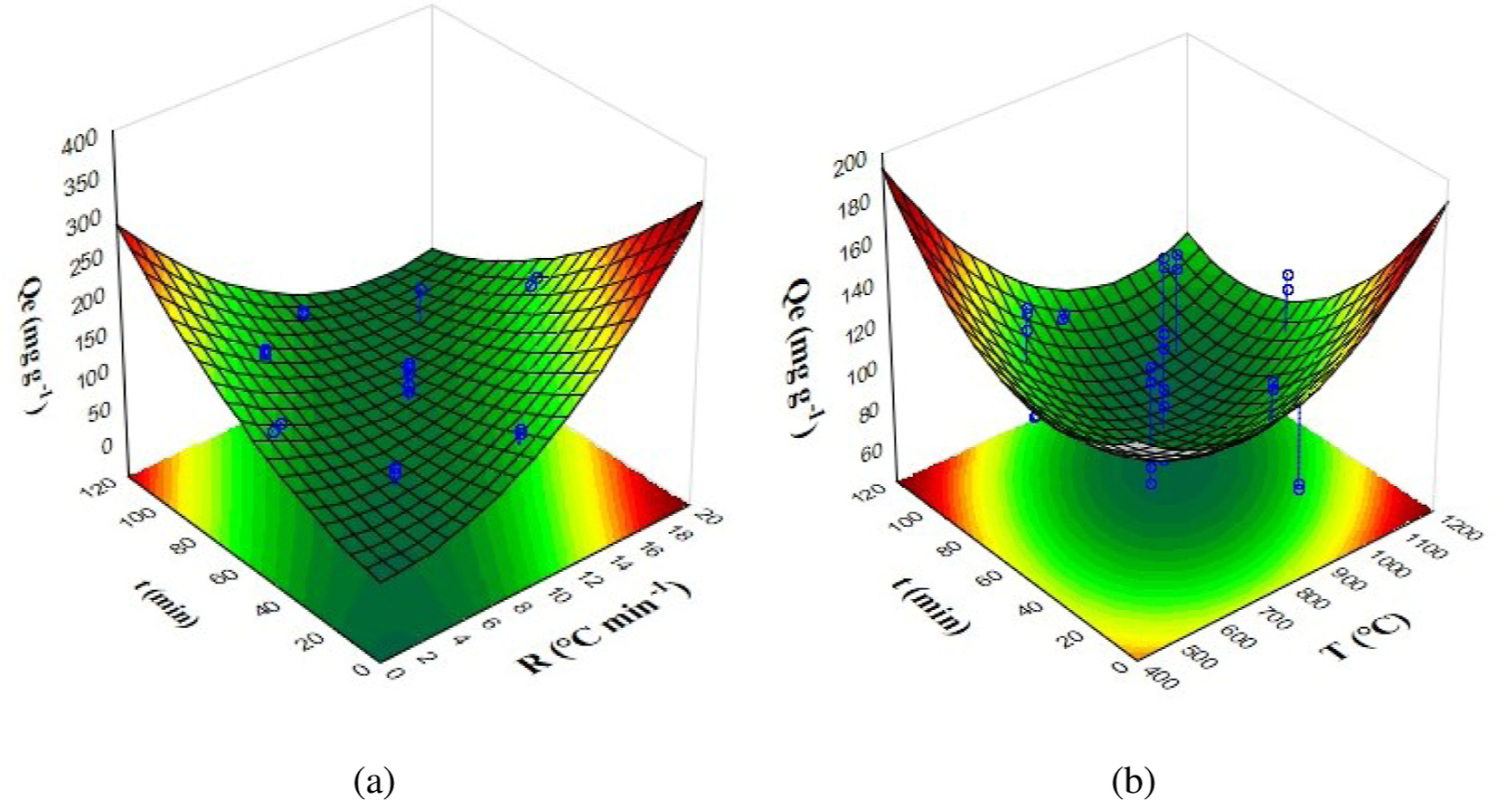
Response surface for CCD1: (a) interaction between heating rate (*R*) and time (*t*), and (b) interaction between temperature (*T*) and time (*t*) on the CV adsorption capacity (*Q*_e_).

Overall, the CCD1 revealed that Run 15 (*T* = 800 °C, *R* = 18.41 °C min^–1^, *t* = 60 min) was the carbonization condition that led to the highest CV dye adsorption capacity, with a *Q*_e_ of 155.45 mg g^–1^. To validate the results and assess the reproducibility of the carbonization process, the same condition was repeated three times, yielding very similar results (*Q*_e_ = 159.36 mg g^–1^), with a relative deviation of 2.5%. These findings confirm the robustness and consistency of the synthesis protocol under optimal thermal conditions. Based on this reproducibility, the identified carbonization parameters from CCD1 were fixed for the next experimental phase, CCD2, which focused on optimizing the precursor composition to further enhance the adsorption performance.

#### CCD2: Effect of the Template-Carbon Precursor Concentrations

In the second experimental design (CCD2), the optimal conditions from CCD1 were fixed to evaluate the effect of the carbon precursor-template concentrations. The experimental results of CCD2 regarding the influence of sucrose concentrations (*C*_S_) and template (*C*_T_) on the adsorption capacity of CV dye are summarized in Table 2, and the individual values are presented in Table S4 (see the Supporting Information), with two replicates for each run. The results indicate that the concentration of the template relative to sucrose influences the adsorption capacity. The Run 11 (*C*_S_ = 23 (% m/V) and *C*_T_ = 17.07 (% m/V)) led to the highest adsorption capacity *Q*_e_ = 223.54 mg g^–1^.

**Table 2:** CCD2: Effect of the Carbon Precursor-Template Concentrations

run	*C*_S_ (% m/V)	*C*_T_ (% m/V)	*Q*_e_ (mg g^–1^)	s.d.^a^
1	13	5	132.94	0.412
2	33	5	20.51	0.689
3	13	15	217.17	2.729
4	33	15	186.71	1.255
5	23	10	119.67	0.046
6	23	10	127.03	2.848
7	23	10	122.20	1.024
8	8.86	10	191.98	3.850
9	37.14	10	74.01	1.509
10	23	2.93	14.81	0.574
11	23	17.07	223.54	4.227

a s.d.: standard deviation.

A variance analysis (ANOVA) was performed to study the adsorption of the crystal violet dye by the MC in relation to the concentrations of carbon precursors (*C*_S_) and the template (*C*_T_). As summarized in Table S5 and the Pareto chart (Figure S3, Supporting Information), most factors, including the linear (*L*) and interaction terms (1L by 2L) were statistically significant, except for the quadratic (*Q*) term of the template concentration (*C*_T_), which was not. Therefore, the behavior of the CV dye adsorption capacity as a function of the sucrose and template (*C*_S_ and *C*_T_) concentration can be expressed by eq 2, which models the adsorption capacity (*Q*_e_) for crystal violet dye.

2 $$Q_{e}\left({mg\;g}^{-1}\right)=231.820-11.808C_{S}+1.295C_{T}+0.4098C_{S}C_{T}+0.081C_{S}^{2}+0.1312C_{T}^{2}$$

The equation illustrates the synergistic interaction between the carbon precursor and template aerosil. The high precision of the quadratic model was confirmed by ANOVA and F-test (see details in the Supporting Information) and can also be observed by comparing the experimental adsorption capacity of 223.54 mg g^–1^ for Run 11, with the predicted value of the model, which was 224.99 mg g^–1^, showing a relative deviation of 0.65%.

Therefore, in Figure 2, the response surfaces for CCD2 are shown, which demonstrate the interaction between the template-sucrose factors and the adsorption capacity of the crystal violet dye. The adsorption capacity may decrease with lower concentrations of the template (*C*_T_). This trend occurs because higher template concentrations enhance the porosity and surface area, providing more active sites for adsorption. Consequently, dye molecules diffuse more effectively into the mesoporous carbon internal structure. As a result, the adsorption capacity increased when the template concentration was high and the carbon precursor concentration was low. In contrast, an excess of sucrose (*C*_S_) significantly reduced adsorption capacity, likely due to pore blockage or reduced template effectiveness, which hindered proper pore formation.

**Figure 2:**
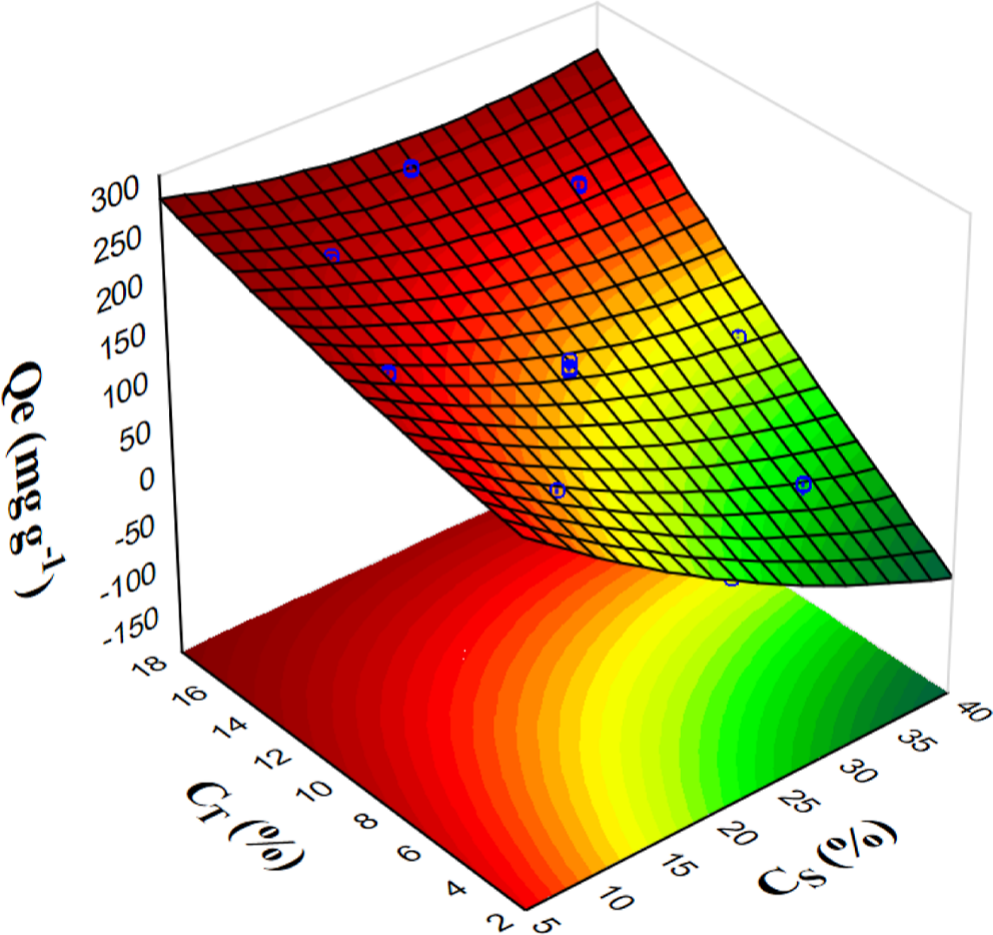
Response surface for the CCD2 for the CV adsorption capacity (*Q*_e_) as a function of the concentrations of the carbon precursor (*C*_S_) and template (*C*_T_) concentrations.

Overall, Run 11 exhibited the highest adsorption capacity *Q*_e_ of 223.54 mg g^–1^ under conditions of *C*_S_ = 23% (m/V) and *C*_T_ = 17.07% (m/V). To confirm the reproducibility of the process, the test was repeated twice under the same conditions, resulting in a consistent adsorption capacity of 221.34 mg g^–1^ with a relative deviation of 1.98%. This demonstrated the reproducibility of the experiment.

By comparing the adsorption efficiency of the optimized mesoporous carbon with various carbon adsorbents reported in the literature for crystal violet dye, as detailed in Table 3, it becomes evident that the mesoporous carbon developed under optimal conditions in this study (CCD2-R11) exhibited a notably high adsorption capacity of 223.5 mg g^–1^ at 30 °C with an initial dye concentration of 100 mg L^–1^. This value is comparable to or exceeds that of many mesoporous and activated carbon-based adsorbents reported in the literature, particularly given the moderate concentration utilized. For example, while nitrogen-doped mesoporous carbon achieved a slightly higher capacity (243.9 mg g^–1^), it was tested at a substantially higher concentration (300 mg L^–1^), which enhances the uptake potential. Similarly, MCM-41, a traditional mesoporous silica, demonstrated lower performance (114.2 mg g^–1^) despite being tested at a higher CV concentration (250 mg L^–1^). Notably, activated carbon derived from *Moringa oleifera* (MO-AC) showed a high adsorption capacity of 469.55 mg g^–1^, although this was realized at a much higher dye concentration (750 mg L^–1^), which may inflate comparative performance assessments. Overall, our material presents a strong balance between the adsorption capacity and ease of synthesis, making it an attractive candidate for practical treatment of micropollutants under moderate operational conditions. Additionally, other conventional activated carbons, such as PA-AC and SA-AC, exhibited significantly lower adsorption capacities (below 25 mg g^–1^), underscoring that the optimized synthesis methodology developed herein yields highly efficient materials even in the absence of chemical activation. This comparison highlights the competitiveness of the MC adsorbent and affirms the efficacy of the optimization strategy employed to tailor the textural properties.

**Table 3:** Overview of Adsorption Capacities of Various Carbon-Based Adsorbents for CV Dye Removal at Specified Conditions

adsorbents	*T* (°C)	*m* (mg)	*C*_0_ (mg L–1)	*Q*_e_ (mg g–1)	ref
nitrogen-doped mesoporous carbon (ND-MC)	30	50	300	243.9	^29^
pulasan fruit peel activated carbon (PUP-AC)	30	90	200	98.9	^30^
Moringa oleifera activated carbon (MO-AC)	∼25	100	750	469.55	^31^
mesoporous silica (MCM-41)	30	20	250	114.2	^32^
phosphoric acid activated carbon (PA-AC)	∼28	100	40	18.5	^33^
sulfuric acid activated carbon (SA-AC)		25		24.45	^33^
mesoporous carbon (CCD1)	30	20	100	155.4	this study
mesoporous carbon (CCD2)				223.5	this study

The findings from CCD1 and CCD2 improved the mesoporous carbon synthesis method to enhance the crystal violet dye adsorption capacity. This two-stage optimization strategy proved to be highly effective as it systematically improved both the thermal treatment parameters and the template-carbon precursor concentration. The optimized parameters (*T* = 800 °C, *R* = 18.41 °C min^–1^, *t* = 60 min; *C*_S_ = 23% m/V, *C*_T_ = 17.07% m/V) effectively enhance the textural properties and adsorption capacity of the mesoporous carbon (MC). When compared to other adsorbents reported in the literature (Table 3), the material synthesized in this study demonstrated one of the highest adsorption capacities for CV dye, even under more demanding conditions. These findings underscore the robustness and efficacy of the RSM-CCD approach implemented, emphasizing its potential as an effective instrument for the design of adsorbent materials with environmental applications.

### Mesoporous Carbons Characterization

#### Thermogravimetric Analysis (TGA-DTG)

The thermal analysis aimed to evaluate the decomposition and thermal stability of MC, as illustrated in Figure 3. The analysis was performed on three different samples: the gel (sucrose + template), the predried sample, and the Aerosil 380 template.

**Figure 3:**
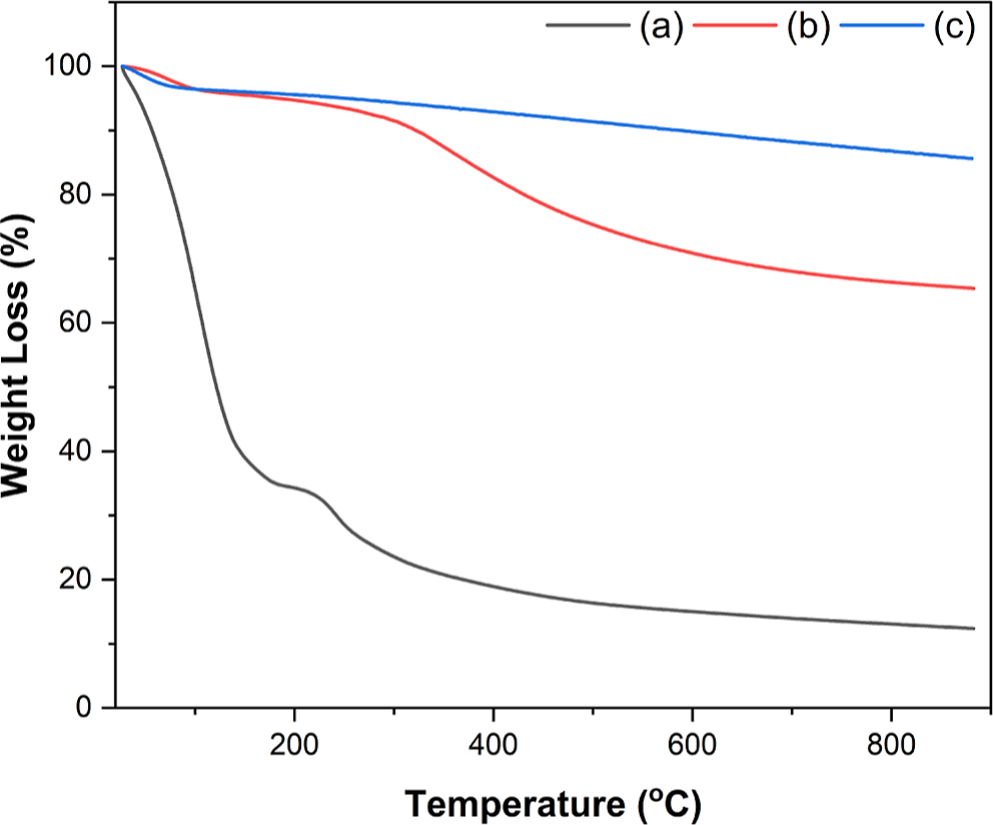
Thermogravimetric analysis of: (a) gel (sucrose solution + template), (b) dried sample, (c) hydrophilic silica Aerosil 380 under the N_2_ atmosphere.

The TGA of the gel revealed two degradation steps (Figure 3a). First, a mass loss of 65.2% was observed, mainly due to evaporation of water and moisture. A second step occurred at 233–900 °C, and the mass loss increased to 87.6%, related to organic compounds, that is, thermally decomposed sucrose, resulting in the formation of carbon structures. The dried sample also showed two degradation steps (Figure 3b), although with a different profile from the gel. During the initial step, the reduction in mass was less pronounced as the majority of water was already removed during the drying stage. The subsequent degradation step commenced at 349 °C, which is a higher temperature compared to that of the gel. This phase is associated with the loss of organic materials, with a mass loss of 34.62% up to 900 °C. In contrast to the gel (Figure 3a) and the dried sample (Figure 3b), the Aerosil 380 template exhibited a single phase of degradation, as shown in Figure 3c. A notable mass loss began at 206 °C, culminating in an overall mass loss of about 14.37%, up to 900 °C. The same template experienced a mass loss of only 1.7%, demonstrating excellent thermal stability up to 800 °C.^34^ Furthermore, the technical datasheet^35^ of the product indicates that hydrophilic fumed Aerosil 380 undergoes a mass loss of no more than 2% when heated to 105 °C for 2 h, most likely due to moisture desorption. This is anticipated considering the high-surface area of Aerosil 380, which enhances the adsorption of water and other molecules from the environment. Overall, it is observed that the TG profile of mesoporous carbon presents a thermal stability of approximately 500–900 °C.

#### Scanning Electron Microscopy and Energy-Dispersive X-ray Spectroscopy (SEM-EDS)

The surface morphology of the mesoporous carbon was analyzed and is illustrated in Figure 4. Although porosity is not directly discernible at this level of magnification, distinct differences in surface morphology were observed between the mesoporous carbon (MC) obtained after leaching and the templated nonleached carbon (TCNL) sample. Figure 4a shows the MC, showing a highly irregular and fragmented morphology with rough textures and pronounced surface granularity, indicative of the successful removal of silica and the formation of a mesoporous framework. In contrast, Figure 4b illustrates the TCNL sample, which presents a more compact and continuous morphology with smoother, well-defined particle surfaces suggestive of a residual silica template. The increased surface heterogeneity observed in the MC enhances the accessibility of both external and internal active sites. This observation aligns with the findings of Che et al.,^36^ who reported that 3D mesoporous structures with heterogeneous surfaces improve access to active sites and, therefore, enhance material performance in various applications.

**Figure 4:**
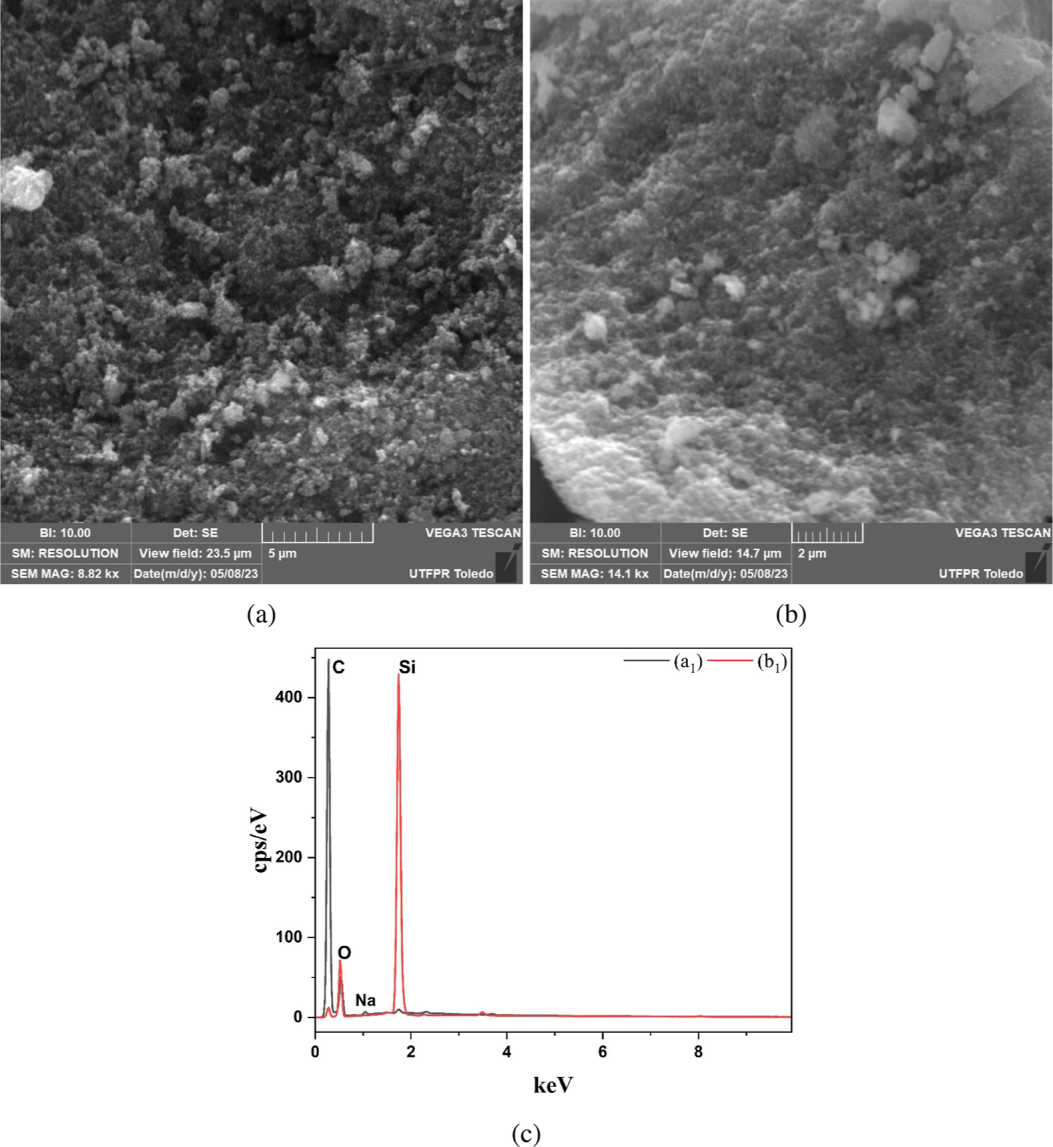
SEM image of (a) mesoporous carbon (MC), (b) templated and nonleached carbon (TCNL), (c) SEM-EDS elemental analysis for: (a_1_) mesoporous carbon (MC), (b_1_) templated and nonleached carbon (TCNL).

Furthermore, energy-dispersive X-ray spectroscopy (EDS) analysis revealed that sodium hydroxide, used as a leaching agent, efficiently removed template Aerosil 380 from the carbon structure. This is evidenced by the EDS spectrum in Figure 4c­(d), which shows a substantial difference in the elemental composition between the nonleached (TCNL) and leached (MC) samples. The leached mesoporous carbon exhibited 83.5% carbon (C), 16.0% oxygen (O), 0.3% sodium (Na), and only 0.1% silicon (Si), confirming the successful removal of nearly 99.6% of the silicon content (from 29.1 to 0.1%) after the leaching process. This drastic reduction is essential for the development of porosity and increased surface area, enhancing accessibility to active sites on both the external and internal surfaces of mesoporous carbon. Similar findings were reported by Liu et al.,^37^ who affirmed that efficient template removal leads to carbon materials with high porosity and extensive surface area.

#### Laser-Induced Breakdown Spectroscopy (LIBS)

The LIBS analysis, as presented in Figure 5, was conducted to evaluate the template leaching. The analysis revealed that sodium hydroxide effectively removed the silica template Aerosil 380 from the MC structure. LIBS analysis confirmed template removal, with the Si line at 288.2 nm serving as a signature of residual Aerosil 380. The characteristic lines of boron at 206.7, 208.9, 249.7, and 345.1 nm were due to the use of boric acid as a binder for the pellet.

**Figure 5:**
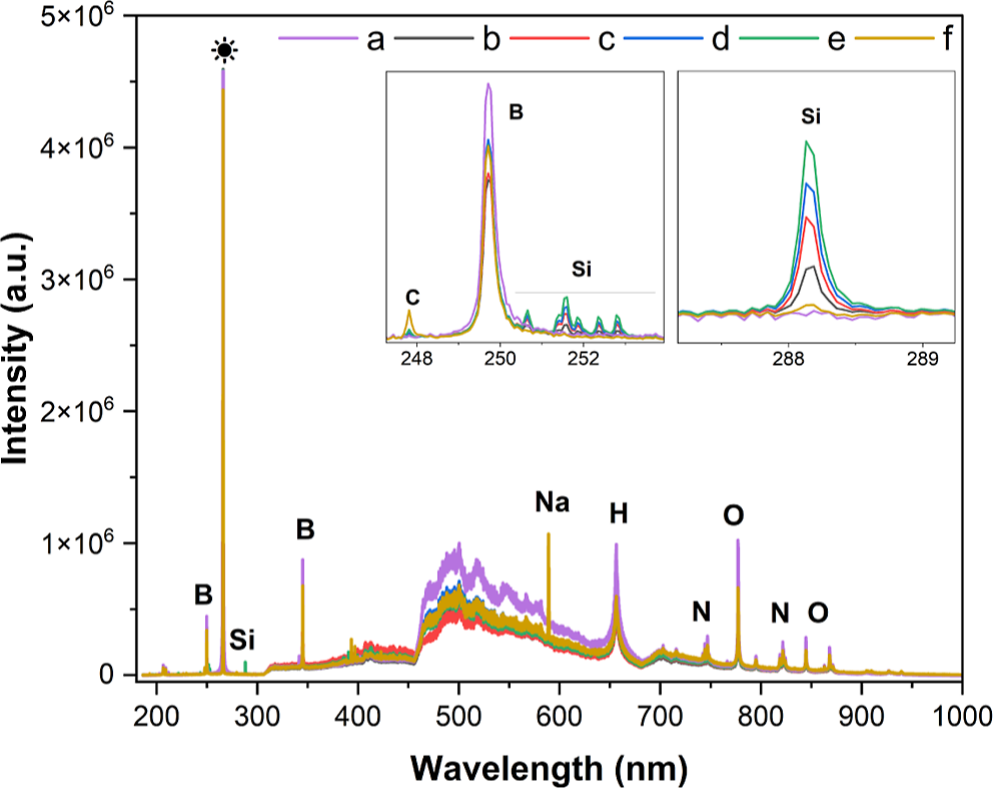
LIBS elemental analysis: (a) boric acid (blank), templated and nonleached carbon: (b) TCNL 2% m/m, (c) TCNL 4% m/m, (d) TCNL 6% m/m, (e) TCNL 8% m/m, and (f) mesoporous carbon (MC) 10% m/m (* symbol denotes the laser line at 266 nm).

According to the calibration curve of TCNL outlined in Figure S8 and Table S8 (see Supporting Information), when TCNL makes up 8% (m/m) of the mixture, it results in a peak area of 22813.9. In contrast, for MC pellet with a concentration of 10 (% m/m), the peak area is 1662.906, quantitatively, with a residual amount of 0.381 (% m/m). This suggests a relatively high template removal efficiency of 96.2 (% m/m). It is important to note that the complete removal was prevented due to the mechanical strength of the carbon, which blocked the leaching agent from thoroughly infiltrating the internal structure of the mesoporous carbon.

It was observed that LIBS measurements identified atoms such as boron (B) from boric acid, nitrogen (N) from the atmosphere, and (*) from the laser line (266 nm). Sodium (Na) was present in insignificant amounts, likely a residue of the leaching agent. Thus, confirming the EDS results for the efficient leaching of the template, carbon (C), oxygen (O), and hydrogen (H) that form hydrocarbons were not quantified because of the low intensity obtained.

LIBS analysis uncovers a comparative difference between residues within the mesoporous carbon structure, where EDS analysis shows the presence of the Si residue at 0.1 (% m/m) within the structure. In contrast, the LIBS spectra indicate 0.381 (% m/m) silicon (Si). This variation is attributed to the sensitivity of the methodology. Despite the numerical variation, both results indicate a very low residual silica content (<0.4%), confirming the effectiveness of the NaOH leaching step. The SEM-EDS is considered a semiquantitative elemental analysis. In particular, LIBS is a quantitative analytical technique that is capable of providing accurate elemental concentrations throughout the bulk of the material. Moreover, for the LIBS analysis, a calibration curve was performed as presented in Figure S8 (provided in the Supporting Information). Hence, it provides reliable quantitative results. In this context, the LIBS result is considered the most representative technique for determining the silica content of the bulk material. Even so, overall, both methods verify the effectiveness of NaOH in removing the template.

#### High-Resolution Transmission Electron Microscope (HR-TEM) Analysis

The HRTEM images of mesoporous carbon show a predominantly irregular morphology with occasional graphitic layer contrast, as shown in Figure 6. This pattern is observed at high resolution (20 nm, Figure 6a) and low resolution (200 nm, Figure 6b). Such morphology may result from the synthesis route and the nature of the template employed, as similar disorganized pore structures of mesoporous carbon have been reported in the literature for templates such as Aerosil 90 and 380,^18^ CaCO_3_,^38^ and KOH.^39^ These HRTEM observations align well with the XRD data, which exhibited broad (002) and (100) reflection features characteristic of turbostratic carbon with limited stacking coherence.^40,41^ The presence of curved and misaligned graphitic layers further confirms the formation of a disordered mesoporous carbon framework.

**Figure 6:**
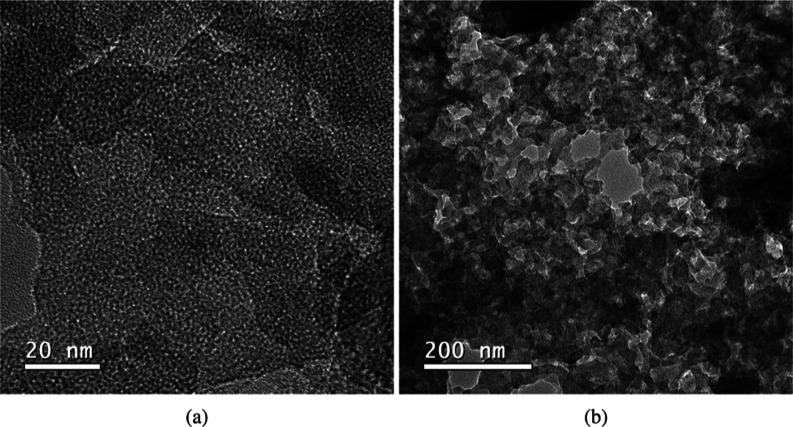
HR-TEM analysis of the mesoporous carbon (MC) at: (a) 20 nm and (b) 200 nm.

#### Textural Parameters—Physisorption of *N*_2_

*N*_2_ physisorption analysis was carried out to assess the textural properties of the mesoporous carbons (MC) at the optimal experimental conditions of CCD1-R15 (Run 15: *T* = 800 °C, *R* = 18.41 °C min^–1^, *t* = 60 min) and CCD2-R11 (Run 11: *C*_S_ = 23% m/V and *C*_T_ = 17.07% m/V). The behavior of nitrogen adsorption and desorption isotherms is shown in Figure 7a, which exhibits a Type IV isotherm according to the IUPAC definition.^42^ This type of isotherm may suggest mesoporosity with associated micropores in its structure due to the hysteresis at lower pressures (*p*/*p*_0_ < 0.04). Similar behavior of the isotherm was also evaluated with mesoporous carbon synthesized from the carbon precursor of phenolic compounds.^43^

**Figure 7:**
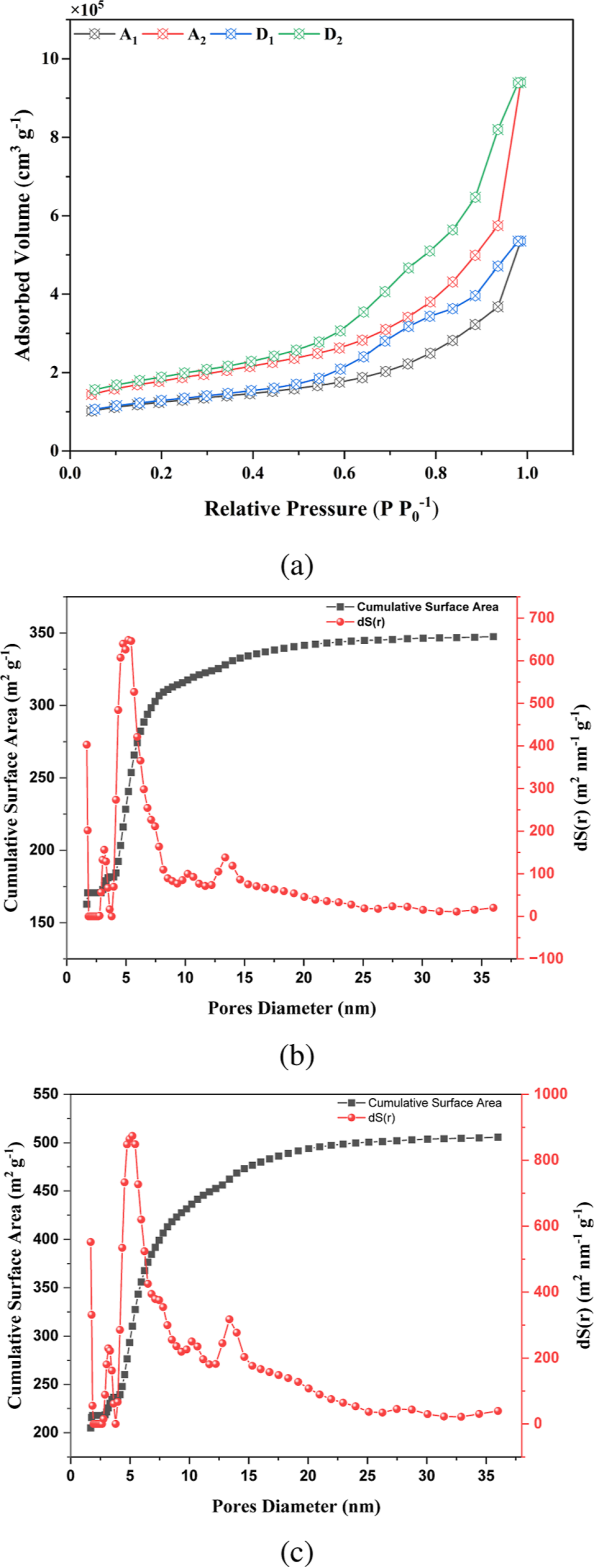
Textural parameters of the MCs: (a) N_2_ physisorption isotherms (CCD1-R15: A_1_—adsorption, D_1_—desorption; CCD2-R11: A_2_—adsorption, D_2_—desorption); and pore size distribution for: (b) CCD1-R15 and (c) CCD2-R11.

The textural parameters outlined in Table 4 reveal a well-developed porosity structure of the mesoporous carbons under the optimized conditions. For instance, the surface area of 417.1 m^2^ g^–1^ for the CCD1-R15 increased to 607.8 m^2^ g^–1^ for the CCD2-R11, representing a 45.73% increase, while the pore diameter increased from 7.96 to 9.60 nm, which is equivalent to a 20.60% increase in the volume of pores. In addition, one may notice a contribution of micropores to the total area of the MCs.

**Table 4:** Textural Parameters of the Mesoporous Carbons (MC) at the best conditions of each CCD

samples	surface area (m^2^ g^–1^)			pores distribution (cm^3^ g^–1^)		
	*A*_ext_	*A*_micro_	*A*_total_	*V*_micro_	*V*_total_	*D*_p_ (nm)
CCD1-R15	257.64	159.46	417.10	0.0812	0.830	7.96
CCD2-R11	442.66	165.16	607.83	0.0844	1.458	9.60

In Figure 7, the pore size distributions in terms of surface area for the optimal experimental conditions of CCD1-R15 and CCD2-R11 are presented. One may notice a more intense peak of pore size close to 5 nm for both samples, evidencing the mesoporous feature of the MCs. Particularly, the peak occurred at 5.4 nm for CCD1-R15 and a slightly smaller value was observed for CCD2-R11, namely, 5.3 nm. Complementarily, the pore size distribution as a function of the pore volume is presented in Supporting Information (see Figure S9). In the best experimental conditions of CCD1 (Figure S9a in Supporting Information), a bimodal pore volume distribution with two relevant peaks can be observed, wherein the most intense peak at 5.44 nm (ranging from 3.7 to 8.6 nm) and another less intense peak at 13.4 nm (between 12.3 and 15.3 nm) are observed. The same peaks can be seen in the distribution of the pores in relation to the surface area in Figure 7b, with the same peak position of 5.4 nm; however, the second peak is much smaller. This happens because the surface area of bigger pores is less relevant than that for smaller pores. In turn, the volume of bigger pores significantly impacts over the total volume of the material. Moreover, a distinct truncated peak with an area of approximately 2 nm is visible, suggesting the presence of micropores. It should be noted that the N_2_ physisorption technique is not suitable for measuring pores smaller than 2 nm. In general, one may conclude that the material is mesoporous and has micropores in its composition.

By examining the results of CCD2-R11 as a function of the pore volume (see Figure S9b in the Supporting Information), one can notice a similar profile to the CCD1-R15 optimized MC with a more intense peak of the secondary peak (at 13.4 nm) when compared to the main peak, which takes place at 5.4 nm, reaching almost the same intensities (1.3 and 1.2 cm^3^ g^–1^), respectively. This is likely due to the increased concentration of the template, which promotes the formation of pores with higher intensity. The same behavior is seen in the distribution for the area (Figure 7c), where the peaks are located at the same positions but with a much higher intensity for the 5.4 nm peak. Similarly, a truncated peak is observed in the micropore range (≈2 nm), which is also more intense than the MC of the CCD1-R15 condition. Overall, it was possible to improve the textural properties of the mesoporous carbons in terms of thermal conditions (CCD1) and carbon-template concentrations (CCD2), resulting in a micromesoporous material with high porosity.

By comparing the pore size of the mesoporous carbons (MCs) with the target adsorbate, one may evaluate the possibility of steric effects and diffusional restrictions. According to Tang et al.,^44^ to achieve efficient adsorption of organic molecules, the diameter of the pores (*D*_p_) should range from 1.7 to 6 times the molecular diameter of the adsorbate (*D*_mol_), as given by eq 3

3 $$R_{p/mol}=\frac{D_{p}}{D_{mol}}$$

where *D*_p_ is the pore diameter of the material (adsorbent), and *D*_mol_ is the diameter of the molecule (adsorbate).

The literature reports that this enhanced adsorption performance due to tailored pore size is known as the “pore-filling effect”, and it can be responsible for the adsorption mechanism along with other intermolecular interactions that may occur for one adsorbate–adsorbent system (e.g., hydrogen bonding, π–π stacking interaction, electrostatic interaction, van der Waals, etc.). In this way, the crystal violet dye has an average diameter of 1.56 nm, which was determined by molecular dynamics simulations using the MMFF94 energy minimization method, as reported by Sausen et al.^45^ This was carried out using MarvinSketch 17.28.0 JSmol software (3D render engine). Therefore, since the most relevant peak in the pore distribution of CCD2-R11 (see Figure 7c) was 5.44 nm, it resulted in a *R*_p/mol_ of 3.63. This value is within the ideal range for the occurrence of the pore-filling effect.

The optimized mesoporous carbons exhibited improved textural properties, making them desirable for use as an adsorbent for the crystal violet dye. This is attributed to its remarkable porosity and tuned pore size and distribution, which facilitates more effective interactions with the molecule. The enhanced textural characteristics account for the high adsorption capacity of the crystal violet dye, as observed for the CCD1-R15, a *Q*_e_ of 155.45 mg g^–1^, and for CCD2-R11 a *Q*_e_ value of 223.54 mg g^–1^. This represents approximately 39.62% of improvement in the adsorption capacity, which is very close to the increase in the total surface area (see Table 4). It is well established that adsorption capacity is directly correlated with surface area, which explains the observed correlation.^46,47^

## Materials and Methods

### Materials

Sucrose (C_12_H_22_O_11_) P.A. grade (*Êxodo Científica*) was used as a carbon source. Hydrophilic fumed silica Aerosil 380 with a surface area of 380–410 m^2^ g^–1^ and a purity of 99.8%, obtained from EVONIC Aerosil, was used as a template. As the leaching agent, sodium hydroxide microbeads (NaOH) P.A. (NEON) were used.

### Synthesis of Mesoporous Carbons (MC)

The synthesis of the mesoporous carbon was based on the procedure as previously reported elsewhere.^18,48–50^ A sucrose precursor solution was initially prepared by mixing a certain amount of sucrose (% m/V) with 50 mL of distilled water. Subsequently, Aerosil 380 (% m/V) was added to the sucrose solution and stirred with a glass rod for 10 min until a homogeneous gel was formed.^2^ The resulting mixture was subsequently placed in crucibles and dried in an oven to eliminate excess water. After drying, the sample was placed in a furnace under vacuum (550 mmHg). Nitrogen (*N*_2_) flow (*Q*N__2__) was then introduced to restore atmospheric pressure, followed by a constant flow of 100 mL min^–1^ to initiate carbonization. After carbonization, 2 M sodium hydroxide (NaOH) was performed to remove the template Aerosil 380 by the leaching process, and the product was oven-dried to remove the water. Finally, mesoporous carbon (MC) was obtained. This process is illustrated in the diagram shown in Figure 8.

**Figure 8:**
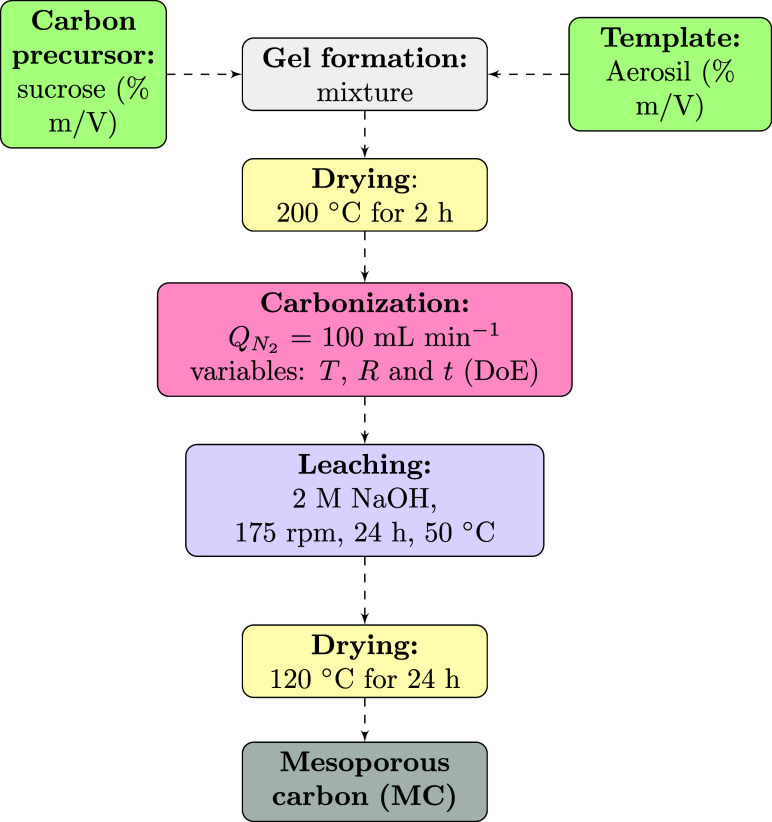
Mesoporous carbons (MCs) synthesis route.

### Mesoporous Carbon Synthesis Optimization

An optimization of the mesoporous carbon synthesis conditions was performed using the sequential design of experiments (DoE) and the response surface method (RSM), where two central composite designs (CCD) were used to evaluate the influence of the conditions of the synthesis process. Initially, the thermal parameters of carbonization (CCD1) were studied with three variables (factors): temperature (*T*), heating rate (*R*), and time (*t*). In this set of experiments, the Aerosil 380 template and the concentration of sucrose precursor were kept constant at 10 and 23% m/V, respectively, in a total of 17 runs. Subsequently, at the best experimental conditions of the CCD1, a two-variable design named CCD2 was used to evaluate the concentrations of the carbon precursor (sucrose) (*C*_S_ % m/V) and the template Aerosil 380 (*C*_T_ % m/V). The CCD2 was carried out in a total of 11 runs. The specifications of the variables, conditions, and levels for both experimental designs are shown in Table 5. Each of the experimental designs was replicated twice. In both cases (CCD1 and CCD2), the adsorption capacity (*Q*_e_) for crystal violet (CV) was the measured output (response variable).

**Table 5:** Specification of Levels and Variables for the Sequential CCDs in the Optimization of Mesoporous Carbon Synthesis

CCD1: effect of carbonization thermal parameters						
factors	codes	levels				
		–α (−1.68)	–1	0	1	α (1.68)
temperature (°C)	*T*	463.64	600	800	1000	1136.37
heating rate (°C min^–1^)	*R*	1.59	5	10	15	18.41
time (min)	*t*	9.55	30	60	90	110.45

CCD2: effect of the carbon precursor-template concentrations						
factors	codes	levels				
		–α (−1.41)	–1	0	1	α (1.41)
sucrose (% m/V)	*C*_S_	8.86	13	23	33	37.14
Aerosil 380 (% m/V)	*C*_T_	2.93	5	10	15	17.07

### Adsorption Assays

Adsorption tests were performed using 20 mg of the adsorbent with standardized granulometry in the range of 14–48 mesh (approximately 1.045 mm) in a volume of 50 mL of a crystal violet dye solution with a concentration of 100 mg L^–1^ at a pH of approximately 5.4. The tests were carried out in the batch system in an orbital shaker at 30 °C and a stirring speed of 150 rpm for 24 h. The samples were then collected and analyzed by UV–vis spectroscopy with a dilution factor of 1:5 at the wavelength of maximum absorbance (584 nm). The adsorption capacities *Q*_e_ (mg g^–1^) were calculated using the mass balance equation given by eq 4

4 $$Q_{e}=\frac{\left(C_{i}-C_{f}\right)V}{m}$$

where *C*_i_ and *C*_f_ denote the initial and final concentrations of the dye in solution (mg L^–1^), *V* represents the solution volume (L), and *m* is the mass of the adsorbent (g).

### Statistical Analysis

Analysis of variance (ANOVA), effect estimates, normal probability, residual analysis, Pareto chart, and response surface methodology (RSM) were all conducted using *software Statistica 14.1.0.8* based on the experimental data from the responses from CCD1 and CCD2. The experimental response models for both designs (CCD1, CCD2) were assessed using a quadratic model given by eq 5.

5 $$Y=\beta_{0}+\sum_{i=1}^{k}\beta_{i}X_{i}+\sum_{i=1}^{k}\beta_{ii}X_{i}^{2}+\sum_{i=1}^{k}\sum_{j=i+1}^{k}\beta_{ij}X_{i}X_{j}$$

where *Y* is the response variable, that is, the adsorption capacity of the CV dye (*Q*_e_), β_0_ is the intercept term, *X*_*i*_ is the encoded variables, β_*i*_ is the linear coefficients for *X*_*i*_, β_*ii*_ is the quadratic coefficients for *X*_*i*_^2^, β_*ij*_ is the interaction coefficients for *X*_*i*_ *X*_*j*_, and *k* is the total number of factors.

The *Shapiro–Wilk* test was applied to the experimental data to assess the normality of the data distribution, with a confidence level of 95% (α = 0.05). Furthermore, a procedure was used to identify and remove *outliers* when it was necessary. After removing the discrepant data, the statistical analysis was carried out again with the updated data.

### Mesoporous Carbons Characterization

The optimized mesoporous carbons (MCs) were characterized by using physical, chemical, textural, and structural analysis techniques. The static laser scattering (SLS) technique (model LA960 from HORIBA Scientific) was used to measure the particle size distribution of mesoporous carbon using the wet method with a carbon refractive index of 1.92 in water, stirring, and circulation velocities of 3. Powder X-ray diffraction (XRD) (model SmartLab SE 3 kW from Rigaku) was performed using the Cu Kα line (1.5418 Å), a nickel filter with a glass sample holder. The experiments were performed using a voltage of 40 kV and 30 mA, with scanning at a rate of 0.05° min^–1^ in the range of θ–2θ (°) from 5 to 70 in a Bragg–Brentano (BB) geometry. Thermogravimetric analysis (TGA-DTG) was performed on gel samples (i.e., sucrose/template solution), dried sample, and Aerosil 380 template using a thermogravimetric analyzer (Shimadzu). The experiments utilized a 15 mg sample under a controlled N_2_ atmosphere (flow rate of 20 mL min^–1^), with the temperature increasing from 30 to 900 °C at a rate of 20 °C min^–1^.

Scanning electron microscopy coupled with energy-dispersive X-ray spectroscopy (SEM-EDS, model VEGA 3 from TESCAN) was used to analyze the surface topography of the mesoporous carbon and perform semiquantitative analysis of the elemental composition and leaching efficiency. Samples were coated with a Au–Pd layer, and images were taken using a secondary electrons detector. EDS mapping was conducted with a power of 30 keV, and six different surface regions were analyzed. The analysis was carried out for templated and nonleached carbon (TCNL) and mesoporous carbon (MC). High-resolution transmission electron microscopy (HRTEM-JEOL) was used to examine the structure of the mesoporous carbon pore network. The experiment was carried out with an accelerating voltage of 200 keV and an optimal Scherzer defocus. The sample was prepared by depositing a dilute isopropyl alcohol solution on a 400 mesh copper grid coated with carbon TED PELLA, type B, and then kept in an ultrasonic bath for 5 min. The magnification ranged from 800 to 1.5 kx with a scale of 20–200 nm, and the analysis was performed only for mesoporous carbon (MC). The laser-induced breakdown spectroscopy (LIBS) analysis was performed using a J200 Tandem LIBS spectrometer from Applied Spectra. The spectrometer featured a 266 nm laser (25 mJ per nanosecond and pulse width (fwhm) of <6 ns), a six-channel charge-coupled device spectrometer with a spectral range of 190 to 1040 nm and a resolution better than <0.1 nm. The spectra were obtained using a 266 nm laser (25 mJ) operating at 60%, a gate delay of 0.5 μs, a spot size of 50 μm, and operated in the line mode (7 lines with 71 shots by line, totaling 497 spectra). For each data set, all spectra were analyzed and averaged by Clarity Software (version 18.0.1.34, Applied Spectra). The signal was collected with a six-channel charge-coupled device spectrometer with a spectral range of 190–1040 nm. The samples were prepared as pellets with boric acid as a blank, with 0–8 (% m/m) of templated carbon and nonleached (TCNL) and 10 (% m/m) of mesoporous carbon (MC). The obtained spectra were examined utilizing the National Institute of Standards and Technology (NIST) database.^51^ The pore size and surface area analyzer (Quantachrome Instruments Novatouch LX2 model) was utilized to assess the textural parameters. The samples were predegassed at 300 °C for 3 h. The specific surface area was determined using the isotherm parameters following the Brunauer–Emmet–Teller (BET) method (*P*/*P*_0_ = 0.05–0.30),^52^ and the pore size distribution was analyzed using the density functional theory (DFT) method. This analysis was performed exclusively for the optimal parameters identified in the experimental design (DoE).

## Conclusions

This study presents a successful synthesis of mesoporous carbon using sucrose and hydrophilic Aerosil 380 via a hard templating method. Through a two-stage optimization strategy based on the response surface methodology (CCD1 and CCD2), optimal conditions were established for both carbonization parameters (*T* = 800 °C, *R* = 18.41 °C min^–1^, and *t* = 60 min) and precursor-template concentrations (*C*_S_ = 23% m/V, *C*_T_ = 17.07% m/V), resulting in a material with enhanced surface area (607.83 m^2^ g^–1^), pore volume (1.458 cm^3^ g^–1^), and adsorption performance (223.54 mg g^–1^). Detailed physicochemical characterization confirmed the formation of a disordered mesoporous carbon structure, marked by hierarchical porosity and efficient template removal. High-resolution transmission electron microscopy (HRTEM) and X-ray diffraction (XRD) revealed turbostratic carbon features, including curved and misaligned graphitic layers, indicative of structural disorder within the pore network. This disordered framework facilitates dye diffusion and adsorption. Also, the N_2_ physisorption analysis revealed that the optimized MC carbon shows a micromesoporous structure with an average pore width of 9.6 nm. However, a relevant peak of pores at 5.4 nm is within an ideal range of tailored pore size for the molecular dimension of the adsorbate, enabling the pore-filling effect, thus contributing to the high adsorption capacities. Overall, the findings highlight the importance of synthesis control in tailoring carbon architectures and demonstrate the reproducibility of this strategy for the development of advanced porous adsorbents for environmental applications.

## Supplementary Material



## Notes

Detailed optimization analyses of sequential experimental designs (CCD1 and CCD2), including experimental matrices, ANOVA, estimated effects, residual analysis, statistical validation of model fitting and factor significance, complementary characterization by static light scattering (SLS), thermogravimetric analysis (TGA-DTG), X-ray diffraction (XRD), laser-induced breakdown spectroscopy (LIBS), and pore size distribution determined by N_2_ physisorption using the DFT method (PDF)
