# Supplementary material for: Optimization of Mesoporous Carbon Adsorbents from Sucrose and Aerosil 380 Hydrophilic Silica by Hard Templating
Source: ACS Omega. 2025 Aug 18;10(34):38890–901. doi: 10.1021/acsomega.5c04653 (PMC12409577; doi:10.1021/acsomega.5c04653)
Supplement: Supplementary file 1 [file ao5c04653_si_001.pdf]

Supporting Information
Optimization of mesoporous carbon adsorbent from sucrose and Aerosil 380 hydrophilic silica by hard templating

João Baptista,[†,‡] Ricardo Schneider,[†,‡] Kelen Menezes Flores Rossi de Aguiar,[†,‡] Aparecido Nivaldo Módenes,[¶] and Fabiano Bisinella Scheufele[*,†,‡]

†*Federal University of Technology – Paraná - UTFPR, Graduate Program in Chemical and Biotechnological Processes (PPGQB),Rua Cristo Rei,19,Vila Becker, Toledo, 85902-490, Paraná,Brazil*

‡*Federal University of Technology – Paraná - UTFPR, Group of Polymers and Nanostructures(GPAN),Rua Cristo Rei,19,Vila Becker, Toledo, 85902-490, Paraná,Brazil*

¶*Western Paraná State University, Chemical Engineering Department, Materials Laboratory, Rua da Faculdade, 645, Jd. La Salle, Toledo,85903-000, Paraná , Brazil*

E-mail: fabianob@utfpr.edu.br

Mesoporous carbon optimization

The synthesis of mesoporous carbons was optimized using the surface response methodology (RSM) employing the sequential rotational central composite design (CCD). CCD1 focused on analyzing the thermal parameters of the carbonization step, while CCD2 investigated the impacts of the template-precursor concentrations. The Shapiro-Wilk method was used to

examine the experimental data for normal distribution, revealing that CCD1 exhibited an outlier point in experiment 4, while CCD2 exhibited two outlier points in experiments 10 and 16. As a result, CCD1 comprised 33 experimental data points, while CCD2 comprised 20 experimental data points.

CCD1: Effect of thermal conditions

This study investigates the impact of carbonization by examining variables such as temperature (T), rate (R), and time (t). The experimental responses are detailed in Table S 1, and the results of the Analysis of Variance (ANOVA) are presented in Table S 2. Furthermore, the estimated effects are outlined in Table S 3. The Pareto diagram, which illustrates the critical factors, is displayed in Figure S 1, whereas Figure S 2a and Figure S 2b present the normal residual and residual plots, respectively.

Statistical analysis - CCD1

For the CCD1 analysis, examining the ANOVA in Table S 2, we observe an F_{calc} value of 6.711, which is bigger than $F_{tab}(5\ \%,9;\ 24)$ of 2.320, suggesting that the quadratic model accurately reflects the experimental data. In addition, the coefficient of determination is relatively high ($R^2 = 0.72$ and $R^2_{adj} = 0.62$, respectively). It can be concluded that the quadratic model is suitable for the complex system of synthesis steps (impregnation, carbonization, and leaching) that is used as an adsorbent, due to its particulate nature and potential for high heterogeneity.

Besides the factors significance results, the residual distribution was analyzed in Figure S 2a and shows that the behavior follows a normal distribution. This was confirmed by the *Shapiro-Wilk* test, which yielded a p value of 0.121, which is greater than α, indicating that the residual distribution is normal and that there is good agreement between the observed data and the theoretical normal distribution. Table S 2b demonstrates that the distributions are random. The residual analysis reveals a random distribution with a residual error of

Table S 1: CCD1 Matrix: Effect of carbonization thermal conditions - Experimental data (in duplicate)

Run	T (℃)	R (℃ min^{-1})	t (min)	Q_e (mg g^{-1})
1	600	5	30	93.637
2	1000	5	30	47.557
3	600	15	30	141.547
4	1000	15	30	(*Outlier*) 59.363
5	600	5	90	130.627
6	1000	5	90	125.258
7	600	15	90	119.346
8	1000	15	90	48.187
9	800	10	60	63.600
10	800	10	60	93.681
11	800	10	60	64.658
12	463.64	10	60	110.015
13	1136.37	10	60	116.022
14	800	1.59	60	121.223
15	800	18.41	60	153.009
16	800	10	9.55	131.176
17	800	10	110.45	98.983
18	600	5	30	85.770
19	1000	5	30	50.413
20	600	15	30	135.062
21	1000	15	90	60.550
22	600	5	90	127.840
23	1000	5	90	117.657
24	600	15	90	119.327
25	1000	15	90	52.872
26	800	10	60	71.073
27	800	10	60	85.881
28	800	10	60	59.098
29	463.64	10	60	109.513
30	1136.37	10	60	122.691
31	800	1.59	60	113.694
32	800	18.41	60	157.897
33	800	10	9.55	127.975
34	800	10	110.45	95.360

Table S 2: ANOVA of CCD1 for Qe (mg g^{-1}) of CV; R^2=0.7242, R^2 adjusted= 0.6163; 3 factors, 1 block, 33 experiments; MS residual=396.46, significance code: $*** = 0.001$; $** = 0.01$; $*= 0.05$

factors	SS	df	MS	F	p
(1)T (℃)(L)	805.06	1	805.06	2.03060	0.167589
T (℃)(Q)	2445.96	1	2445.96	6.16946	0.020719 *
(2)R (℃ min^{-1})(L)	2445.91	1	2445.91	6.16931	0.020720*
R (℃ min^{-1})(Q)	7321.37	1	7321.37	18.46670	0.000268 ***
(3)t (min)(L)	812.11	1	812.11	2.04838	0.165817
t (min)(Q)	2243.34	1	2243.34	5.65838	0.026058 *
1L by 2L	0.00	1	0.00	0.00000	0.998643
1L by 3L	869.70	1	869.70	2.19364	0.152152
2L by 3L	11752.15	1	11752.15	29.64245	0.000016 ***
Regression	23944.56	9	2660.507	6.71060	
Error	9118.66	23	396.46		
SS Total	33063.22	32			

396.46. The normal and random distributions observed in the experimental data indicate that the assumptions of the ANOVA and the F-test are valid.

Table S 3: Effect estimates for Qe (mg g^{-1}) for CCD1; R^2=.72421; R^2-adjusted:.61629. 1 block, 33 runs; MS residual=396.463, significance code: $*** = 0.001$; $** = 0.01$; $*= 0.05$

factors	effects	std. error	t(23)	p	-95,% cnf.limt	+95,% cnf.limt	coeff.	std.error	-95,% cnf.limt	+95,% cnf.limt
mean/intercept.	74.687	8.09786	9.22306	0.000000 ***	57.935	91.439	74.6871	8.09786	57.935	91.4388
(1)T (℃)(L)	-19.943	13.99488	-1.42499	0.167589	-48.893	9.008	-9.9713	6.99744	-24.447	4.5040
T (℃)(Q)	59.419	23.92206	2.48384	0.020719 *	9.932	108.905	29.7093	11.96103	4.966	54.4525
(2)R (℃ min^{-1})(L)	35.158	14.15483	2.48381	0.020720 *	5.876	64.439	17.5789	7.07741	2.938	32.2197
R (℃ min^{-1})(Q)	106.916	24.87979	4.29729	0.000268 ***	55.448	158.383	53.4578	12.43990	27.724	79.1917
(3)t (min)(L)	-20.140	14.07172	-1.43122	0.165817	-49.249	8.970	-10.0698	7.03586	-24.625	4.4849
t (min)(Q)	57.286	24.08252	2.37874	0.026058 *	7.467	107.104	28.6430	12.04126	3.734	53.5522
1L by 2L	-0.056	32.45167	-0.00172	0.998643	-67.187	67.076	-0.0279	16.22583	-33.594	33.5378
1L by 3L	-48.322	32.62586	-1.48109	0.152152	-115.814	19.170	-24.1610	16.31293	-57.907	9.5849
2L by 3L	-177.650	32.62926	-5.44449	0.000016 ***	-245.148	-110.151	-88.824	16.31463	-122.574	-55.0754

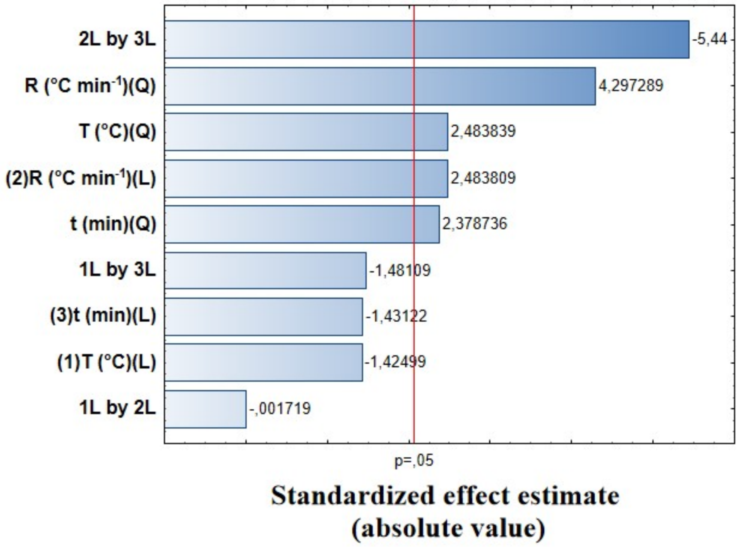

Figure S 1: Pareto Chart of CCD1: Effect of carbonization thermal parameters.

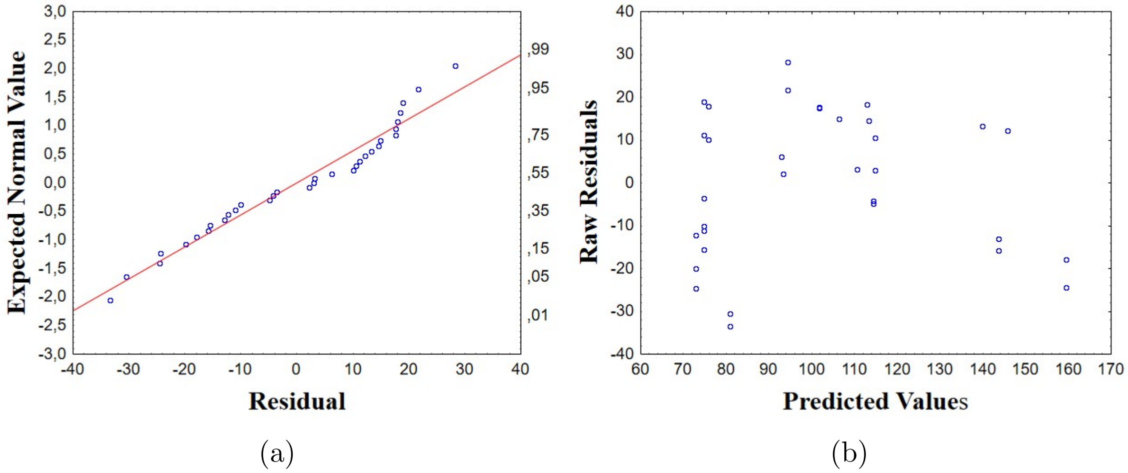

Figure S 2: CCD1 residual analysis – graphs of: (a) normal distribution, (b) residuals vs. predicted values.

CCD2: Effect of the *template*-carbon precursor concentrations

The second experimental design evaluated the impact of template-precursor concentrations. The experimental results are displayed in Table S 4, with the ANOVA results in Table S 5 and the estimated effects of the adsorption capacity (Q_e) in Table S 6. The Pareto chart illustrating significant factors is provided in Figure S 3, and the plots for residual normality and residuals are shown in Figure S 4a and Figure S 4b, respectively.

Table S 4: CCD2 Matrix: Effect of template-precursor concentrations - Experimental data (in duplicate)

Run	Sucrose - C_S (%)	Template - C_T (%)	Q_e (mg g^{-1})
1	13	5	133.35
2	33	5	21.20
3	13	15	214.45
4	33	15	187.97
5	23	10	119.63
6	23	10	124.18
7	23	10	123.23
8	8.86	10	188.13
9	37.14	10	75.52
10	23	2.93	(*Outlier*) 14.24
11	23	17.07	227.76
12	13	5	132.53
13	33	5	19.82
14	13	15	219.90
15	33	15	185.46
16	23	10	(*Outlier*) 119.72
17	23	10	129.88
18	23	10	121.18
19	8.86	10	195.83
20	37.14	10	72.51
21	23	2.93	15.39
22	23	17.07	219.31

Statistical analysis - CCD2

ANOVA analysis, in Table S 5, showed that the F_{test} distribution resulted in F_{calc} being 230.186, which is significantly higher than the F_{tab}(5 %,5; 14) value of 2.958. This ob-

servation ($F_{calc} > F_{tab}$) confirms that the quadratic model suitably and statistically fits the experimental data. Furthermore, the fit determination coefficient R^2 was high ($R^2 = 0.98798$; $R^2_{adj} = 0.98369$), indicating that the model accurately describes the experimental data for the adsorption capacity of the crystal violet dye as a function of the factors studied.

Table S 5: ANOVA of adsorption capacity Qe (mg g^{-1}) of CV for CCD2; R^2=0.98798; R^2 adjusted=0.98369; 2 factors, 1 blocks, 20 experiments; MS residual error=78.897, significance code: $*** = 0.001$; $** = 0.01$; $*= 0.05$

factors	SS	df	MS	F	p
(1)C_S (%)(L)	25081.40	1	25081.40	317.9019	0.000000 ***
C_S (%)(Q)	667.18	1	667.18	8.4564	0.011460 **
(2)C_T (%)(L)	60467.05	1	60467.05	766.4083	0.000000 ***
C_T (%)(Q)	99.28	1	99.28	1.2584	0.280833
1L by 2L	3359.54	1	3359.54	42.5815	0.000013 ***
Regression	90804.52	5	18160.9	230.18598	
Error	1104.55	14	78.90		
Total SS	91909.07	19			

Table S 6: Effect estimates for Qe (mg g^{-1}) for CCD2; R^2=.98798; R^2 adjusted=.98369; 2 factors, 1 blocks, 20 experiments; MS residual=78.896; significance code: $***$ = 0.001; $**$ = 0.01; $*$= 0.05

factors	effects	standard error	t(27)	p	-95% cnf.limt	+95% cnf.limt	coefficient	standard error of coef.	-95% cnf.limt	+95% cnf.limt
mean/intercept	123.6274	3.972319	31.1222	0.000000 $***$	115.1076	132.1471	123.6274	3.972319	115.1076	132.1471
(1)C_S (%)(L)	-79.3036	4.447810	-17.8298	0.000000 $***$	-88.8432	-69.7640	-39.6518	2.223905	-44.4216	-34.8820
C_S (%)(Q)	16.2805	5.598536	2.9080	0.011460 $**$	4.2728	28.2881	8.1402	2.799268	2.1364	14.1441
(2)C_T (%)(L)	133.4516	4.820519	27.6841	0.000000 $***$	123.1126	143.7906	66.7258	2.410259	61.5563	71.8953
C_T (%)(Q)	6.5592	5.847198	1.1218	0.280833	-5.9818	19.1002	3.2796	2.923599	-2.9909	9.5501
1L by 2L	40.9850	6.280790	6.5255	0.000013 $***$	27.5140	54.4560	20.4925	3.140395	13.7570	27.2280

It is evident from Figure S 4a that the experimental data follow a normal distribution. This is further confirmed by the *Shapiro-Wilk* test, which yields a p value of 0.9203, greater than the α value. This implies that the observed distribution is in agreement with the theoretical normal distribution. Furthermore, the residuals in Figure S 4b are randomly distributed with a residual error of 78.897. This implies that the experimental data of CCD2 are normally and randomly distributed.

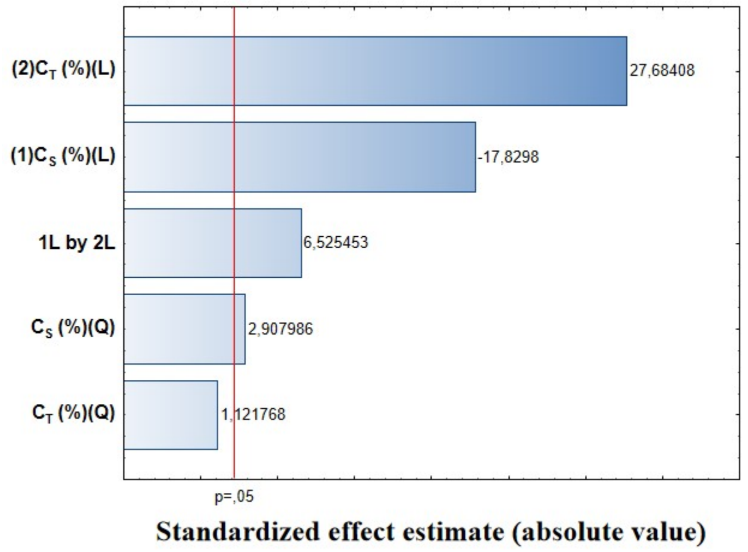

Figure S 3: CCD2 pareto chart of adsorption capacity (Q_e) as a function of C_S and C_T concentrations.

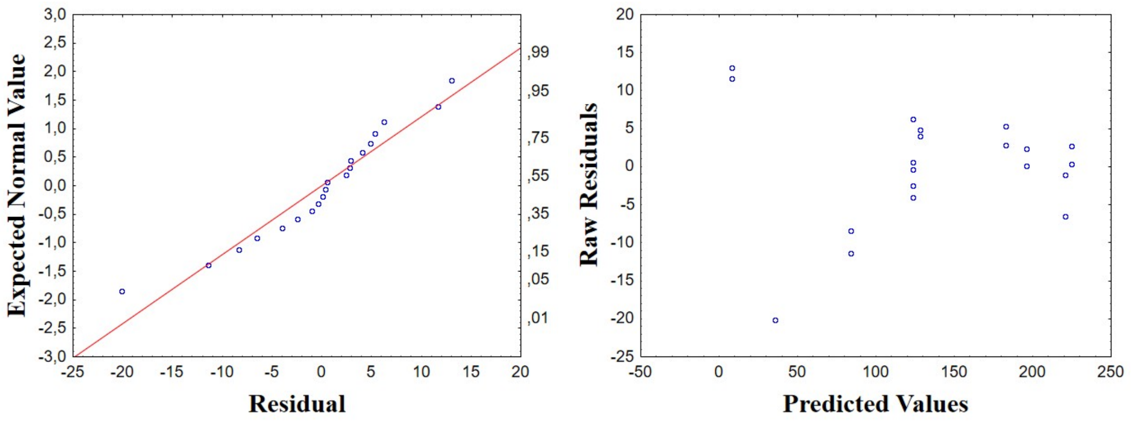

(a) (b)

Figure S 4: CCD2 residual analysis – graphs of: (a) normal distribution, (b) residuals vs. predicted values.

Characterization analyses

Granulometry – Static laser scattering analysis (SLS)

Static laser scattering (SLS) analysis was performed using the wet method to assess the particle size distribution. SLS results showed that the particles had an average diameter of around 1 mm, exhibiting a single-peak distribution. The particle diameters of 558.21 μm, 952.09 μm and 1624.87 μm were identified in the fractions $q_{10\%}$, $q_{50\%}$ and $q_{90\%}$, respectively. The particle size distribution and the cumulative undersize fractions are presented in Figure S 5.

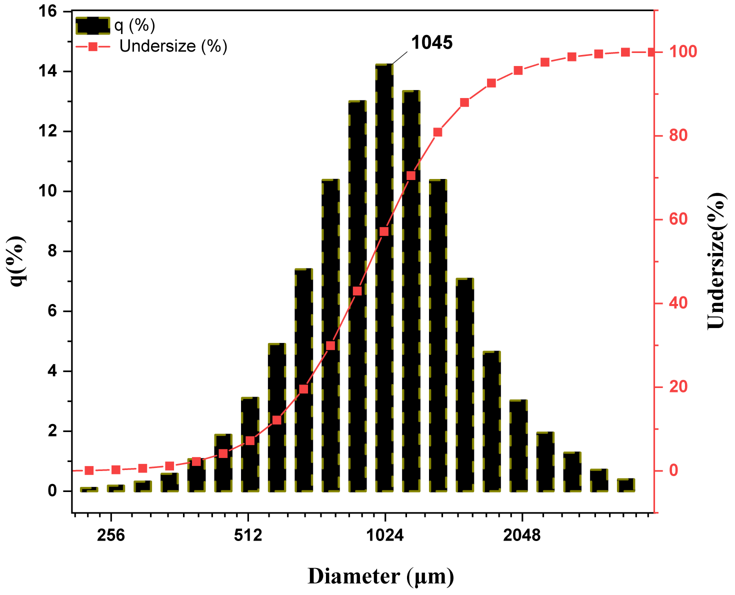

Figure S 5: Mesoporous carbon (MC) granulometry analysis using Static Laser Scattering Analysis (SLS).

Thermogravimetric analysis (TGA-DTG)

Derivative thermogravimetric analyses provide stage decomposition behavior of the carbon precursors and the template. As shown in Figure S 6 and summarized in Table S 7, two distinct stages of mass loss are observed. Stage I, associated with the evaporation of water and volatile compounds, resulted in a significant mass loss of 65.20 % for the gel sample

(containing sucrose and template), whereas the dried sample and Aerosil 380 showed minimal losses of 3.76 % and 3.31 %, respectively, indicating low moisture content. In stage II, which corresponds to the thermal decomposition of organic matter and carbonization, the gel experienced a substantial additional loss of 87.60 %, confirming the degradation of sucrose. In comparison, the dried sample lost 34.62 %, while the Aerosil 380 exhibited a lower loss of 14.37 %, consistent with its inorganic nature and high thermal stability.

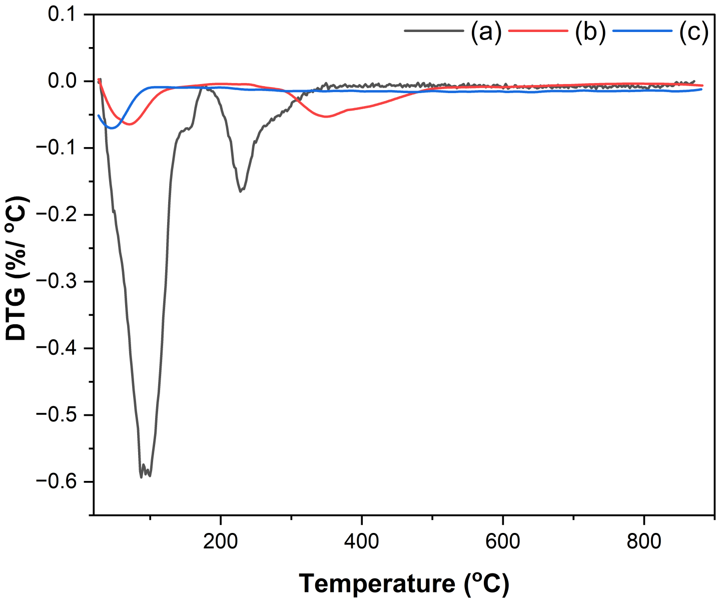

Figure S 6: Derivative thermogravimetric analyses (DTG) of: (a) gel (sucrose solution+*template*), (b) Dried sample, (c) hydrophilic silica Aerosil 380 under the N_2 atmosphere

Table S 7: Mass loss (%) at each degradation stage of the carbon precursors and template.

Samples	Stage I		Stage II	
	T(°C)	Mass loss (%)	T(°C)	Mass loss (%)
Gel (sucrose solution + template)	30-179	65.20	233-900	87.60
Dried sample	30-233	3.76	349-900	34.62
Silica Aerosil 380	30-127	3.31	205-900	14.37

X-ray diffraction analysis (XRD)

X-ray diffraction analysis was conducted on both the mesoporous carbon (a) and the templated non-leached carbon (TCNL) (b), as shown in Figure S 7. The XRD pattern of the

mesoporous carbon (a) displays a broad (002) reflection centered at approximately 22.8 °, indicative of a turbostratic arrangement of graphitic layers with poor stacking order. A secondary, less intense (100) reflection appears near 43.4 °, suggesting limited in-plane structural organization. These broad and low-intensity peaks are characteristic of turbostratic carbon, as previously described by Li et al.[1] and Fujimoto,[2] who attributed such features to misaligned and rotationally disordered graphitic domains. Conversely, the TCNL sample (b), which did not undergo template removal, exhibits markedly lower intensity and a lack of distinct diffraction peaks, reflecting a highly amorphous structure with minimal graphitization. These findings align with established literature on mesoporous carbon materials, which typically report a broad (002) peak between 22–26 ° and a weak (100) diffraction between 43–46 °, consistent with disordered graphite-like structures.[3–5]

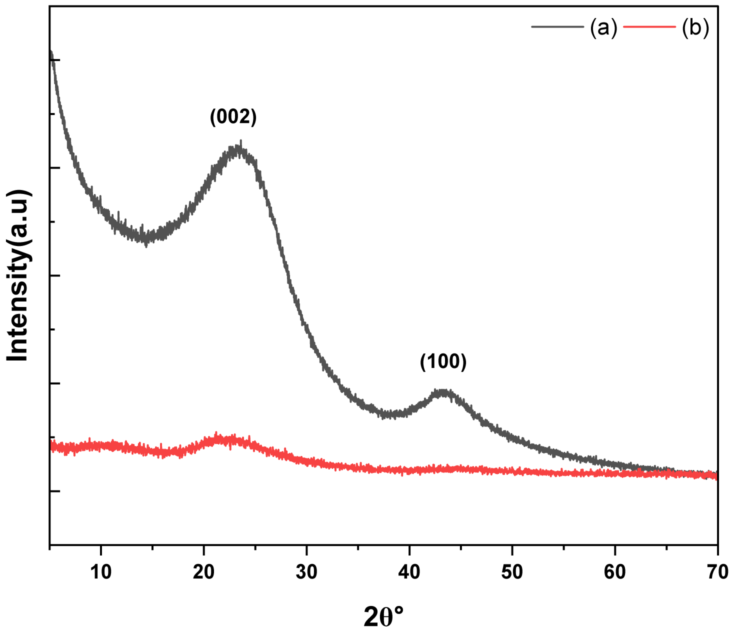

Figure S 7: X-ray diffractogram of: (a) mesoporous carbon (MC), (b) templated and non-leached carbon (TCNL)

Quantification of Si analysis by Laser Induced Breakdown Spectroscopy (LIBS)

The calibration curve presented in Figure S 8 was constructed specifically for unleached carbon samples (TCNL) containing Silicon (Si), prepared with boric acid as the matrix and standardized tablet weight at 100 mg, covering concentrations of 0, 2, 4, 6, and 8 % (w/w) (TCNL vs. Boric Acid). The excellent linear fit ($R^2 = 0.997$) demonstrates that the intensity of the Si peak at 288.2 nm increased proportionally with the increase of the TCNL concentration, confirming the linear relationship between TCNL content and the spectral response obtained by LIBS. Based on this curve, the residual amount of Si in the leached sample denoted mesoporous carbon, prepared with 10 wt % presented in Table S 8, was estimated, resulting in 0.384 % of remaining Si. This corresponds to a removal efficiency of 96.2 %, thus evidencing the effectiveness of the leaching process.

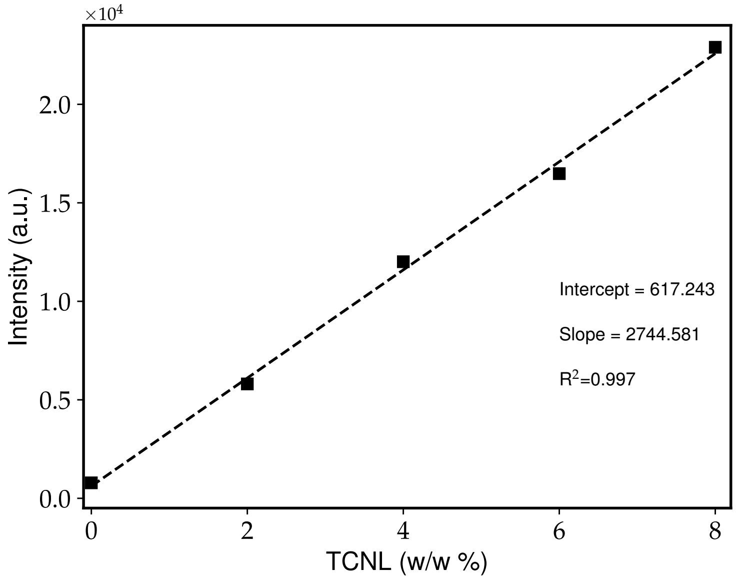

Figure S 8: Calibration curve using 288.2 nm line of Si. Non-leached templated carbon (TCNL) was used as a reference in LIBS analyses.

Table S 8: Amounts of TCNL and relative Si peak intensity at 288.2 nm for calibration curve.

TCNL	MC	Si peak intensity[(a)]	Si non-leached amount[(b)]
(%) m/m		a.u.	(%) m/m
0	-	786.1116	-
2	-	5806.294	-
4	-	12009.94	-
6	-	16480.84	-
8	-	22894.65	-
-	10	1662.906	0.381(96.2 %)[(c)]

[(a)] averaged
[(b)] The removal amount is relative to the sample non-leached
[(c)] Obtained with fit parameters, Figure S8 considering a 100 % MC pellet.

Pore size distribution - Physsisorption of N_2 analysis

Figure S 9 presents the pore structure characteristics of the CCD1-R15 and CCD2-R11 samples based on the DFT method. In both samples, the cumulative pore volume (black squares) steadily increases with pore diameter, indicating a well-developed mesoporous network. For CCD1-R15 (Figure S 9a), the cumulative pore volume reaches approximately 0.75 cm^3 g^{-1}, with a significant fraction of pores centered around 4–10 nm. The red circles exhibits multiple peaks within this range, highlighting a heterogeneous pore distribution. In contrast, CCD2-R11 (Figure S 9b) shows a higher cumulative pore volume exceeding 1.2 cm^3 g^{-1} and a broader pore size distribution, with prominent peaks in the 3–12 nm range, suggesting a more developed and diverse mesoporosity. These results indicate that CCD2-R11 possesses a larger surface area and pore volume, which could enhance molecular diffusion and accessibility to active sites in adsorption applications.

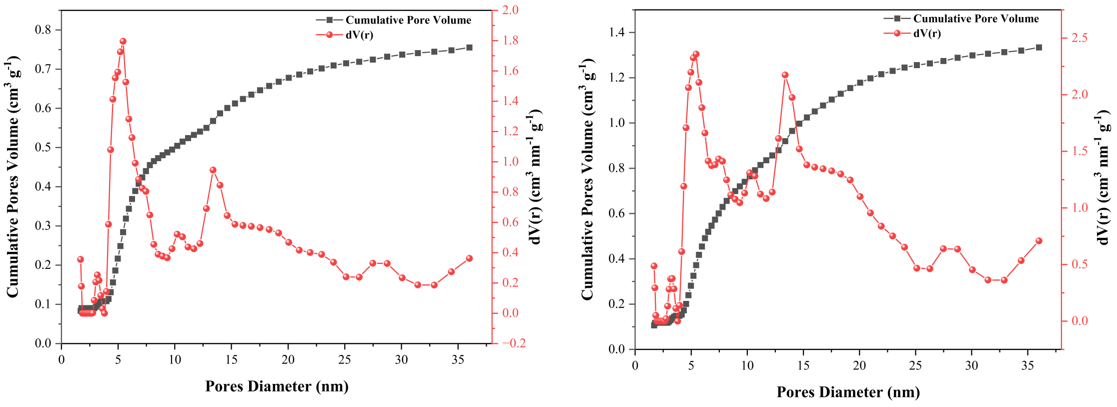

(a) (b)

Figure S 9: Pore size distribution as a function of pore volume (cm^3 g^{-1}) by DFT method for optimal mesoporous carbons samples: (a) CCD1-R15 and (b) CCD2-R11.

References

(1) Li, Z.; Lu, C.; Xia, Z.; Zhou, Y.; Luo, Z. X-ray diffraction patterns of graphite and turbostratic carbon. *Carbon* **2007**, *45*, 1686–1695.

(2) Fujimoto, H. Theoretical X-ray scattering intensity of carbons with turbostratic stacking and AB stacking structures. *Carbon* **2003**, *41*, 1585–1592.

(3) Faisal, M.; Pamungkas, A. Z.; Krisnandi, Y. K. Study of Amine Functionalized Mesoporous Carbon as CO2 Storage Materials. *Processes* **2021**, *9*, 456.

(4) Cao, Y.; Cao, J.; Zheng, M.; Liu, J.; Ji, G. Synthesis, characterization, and electrochemical properties of ordered mesoporous carbons containing nickel oxide nanoparticles using sucrose and nickel acetate in a silica template. *Journal of Solid State Chemistry* **2007**, *180*, 792–798.

(5) Mao, Y.; Duan, H.; Xu, B.; Zhang, L.; Hu, Y.; Zhao, C.; Wang, Z.; Chen, L.; Yang, Y. Lithium storage in nitrogen-rich mesoporous carbon materials. *Energy & Environmental Science* **2012**, *5*, 7950.
